# Reminiscences about kuru and the people of Okapa

**DOI:** 10.1098/rstb.2008.4027

**Published:** 2008-11-27

**Authors:** Adolf Saweri

**Affiliations:** Division of Medicine, School of Medicine and Health Sciences, University of Papua New GuineaPO Box 5623, Boroko, NCD 111, Papua New Guinea

I first heard of ‘de lachende ziekte’ on Dutch newsprint and radio in 1957 while at high school, then promptly forgot about it until my medical school years. My impression of the first patient I saw demonstrated in clinical neurology class was vague. The first visit to Okapa (1964–1965) was bland. The class of eight arrived at 7 in the evening and left for ‘kuru ward round’ at 6 the next morning with a piece of dry scone and lemon tea for breakfast. Kuru ward round was walking up and down mountains seeing patients in their hamlets, and this could take a whole day. This particular round, led by Dr Richard (Dick) Hornabrook, started at 7 in the morning and ended at 3 in the afternoon. The only memory left of that ward round was the exhaustion and painful blistered feet from mountain climbing.

When I returned to Okapa as a rural medical officer (bush doctor) in 1969 the epidemic was on the wane. Kuru had been transmitted to chimpanzees 4 years previously, but the fact only sunk in when Carleton Gajdusek and Michael Alpers on their field visit during the year filled me in.

The primary duties of the ‘bush doctor’ were ensuring that every aid post was operational and that the mobile infant and maternal clinics were conducted regularly, and providing a clinical service at the Okapa station (subdistrict centre). The other duties were playing host to visiting medical specialists and health administrators. It was in this function that I trailed behind Dick Hornabrook when he came to visit kuru patients and so I learned more about the patients and their care (social environment). The Institute of Human Biology (as the Papua New Guinea Institute of Medical Research was then called) maintained an office and flat at Okapa and several field reporters. Once Dick brought Professor Ian MacDonald (from Queen Square, London) for a few days to show him some patients. I had free tutorials in clinical neurology from both of them.

‘Medical tourists’ often dropped in late in the afternoon and asked to see a kuru patient. I generally turned down such requests (i) because the patients were not zoo animals and (ii) owing to the disruption to the daily work programme. An exception was made for Dr Frederick J. Wright of the University of Edinburgh who wanted to see other tropical diseases as well as kuru.

After I left Okapa, I went back again for short visits but never to work. I was kept abreast of the status of kuru through the Director's reports at meetings of the Papua New Guinea Institute of Medical Research Council, of which I have been a member since 1973.

So in truth, I gained more than I gave as service to kuru and Okapa health in general. I learned about the people's care and compassion for family members with incurable illness. They coped ably without aids from organized government or NGO social services. That level of care and commitment can never be repeated in contemporary Papua New Guinea, or the world for that matter.

